# Veterinary vaccine production using bioreactors: scale up study from laboratory and pd up to commercial production

**DOI:** 10.1186/1753-6561-9-S9-P76

**Published:** 2015-12-14

**Authors:** Lídia Garcia, Maria Turon, Marta Comellas, Mercedes Mouriño, Alicia Urniza

**Affiliations:** 1 ZOETIS MANUFACTURING & RESEARCH SPAIN, S.L Pfizer Olot S.L.U., Ctra. Camprodon s/n, La Riba, 17813 Vall de Bianya (Girona), Spain

## Background

Vaccination has proven itself as the most effective tool to control and prevent the disease and to facilitate the safe trade of live animals.

Viral vaccine manufacturing processes present some specific constraints as compared to other biotech products linked to the cell substrate used and to the viral production.

Multiple cell lines are used for productions such as VERO, MDCK, MRC5, BHK, and CHO cells. BHK-21 cells were selected because they are the most commonly used cells for vaccine production.

As the feasibility of transferring the BHK-21 cells growth in microcarriers from a conventional bioreactor to single-use bioreactor (SU) was demonstrated, actually was time to scale-up and verify the flexibility and ease of use of these SU bioreactors enable rapid scale-up without any loss in product quality. This document describes a scaled-up production of a bovine viral vaccine.

Identification of optimal parameters values, testing at intermediary bioreactors scales (2 liters - 50 liters) and application of the new process settings at industrial bioreactor scale of 200 liters were performed. Taking into account that a critical feature of microcarrier-based cell culture is the homogeneity of cell adhesion to the beads, mixing and aeration performance evaluation remain mandatory to ensure robust scale-up of cell culture processes.

The goal of this study was to evaluate the possibility to produce the antigens for animal vaccines manufacturing in a bioreactor SU as alternative to roller bottles without any loss in product quality. Evaluation of COG reduction was done.

## Materials and methods

Selection of appropriate culture conditions can be important to achieve consistent cell culture and virus production across sites and scales. Because characteristics like tank geometry and hardware (impellers, sparger) are not subject to change during scale-up, the scalability from 2L to 200L in the BIOSTAT® STR bioreactor was an easy strategy for our production process.

Several studies were conducted to compare the growth of BHK-21 cells in microcarriers.

Identification of optimal parameters values and testing at intermediary bioreactor scales (2 liters - 50 liters). Application of the new process settings at industrial fermentor scale (200 liters)

For the scale down models, the cultures were conducted with:

- Biostat B plus and Bisotat B (Sartorius-stedim): Univessel® SU 2L biorector and conventional bioreactor with glass vessels of 10L.

- BIOSTAT® CultiBag STR 50 L

- BIOSTAT® CultiBag STR 200 liters

Cultures were regularly sampled to monitor the in-process parameters such as final cell concentration and product yield (TCID50/ml titration). In this study, these performances were evaluated.

Finally, taking into account that for vaccine formulation microcarriers must be eliminated from the viral suspension, filtration through Sartopure PP2 cartridges (from Sartorius Stedim Biotech ) was performed.

## Results

The most important process criterion for evaluating the performance of each size of bioreactor is their ability to support the same level of viral production as in a conventional bioreactor. To evaluate this point, antigen production for a bovine vaccine was produced in the different bioreactors.

**Figure 1:**
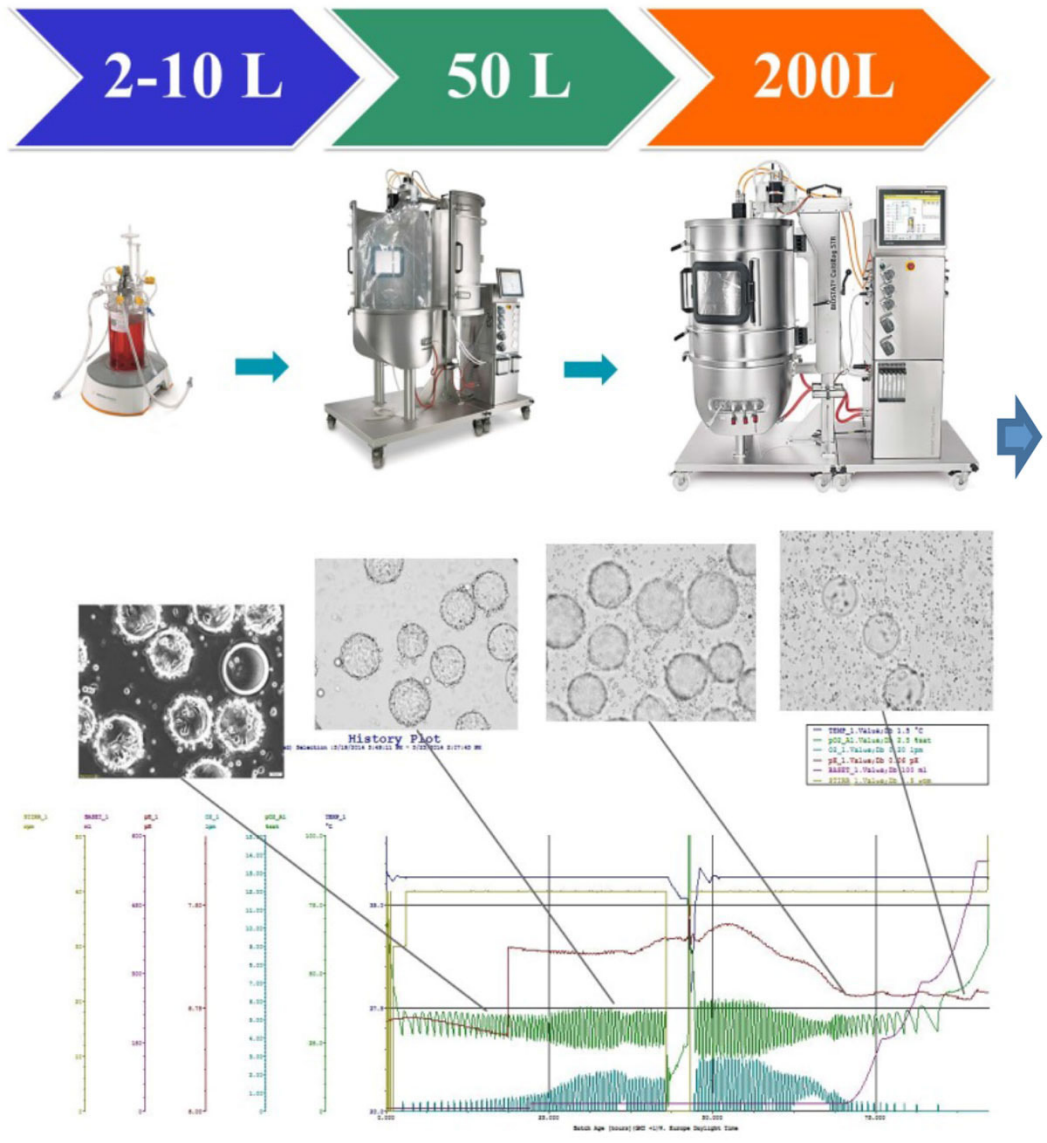
**Results of cell culture and infectivity at 200L scale**.

• Comparable results between 2L, 10L, 50L and 200L concerning cell density, viability, productivity and product quality (Figure 1).

• Bioreactor system allows even equivalent cell growth profiles and equivalent cell population homogeneity on microcarriers.

• Good cell attachment and propagation.

• Very accurate pO2 control in BIOSTAT CultiBag STR 50 and 200L. Good gas transfer capacity necessary to decrease the amount of oxigen needed to mantain the dissolved-oxygen concentration in the set point selected. Good agitation and easy speed calculation according scale-up.

• Viral production obtained with large scale bioreactors was equivalent to the one obtained with the Univessel SU 2L bioreactor (Figure 1).

• One concern for the bovine viral vaccine preparation is how to eliminate microcarriers retaining the cellular debris, several assays filtrating through Sartopure PP2 or PP3 cartridges were performed obtaining no significant drop in virus titer.

**Figure 2:**
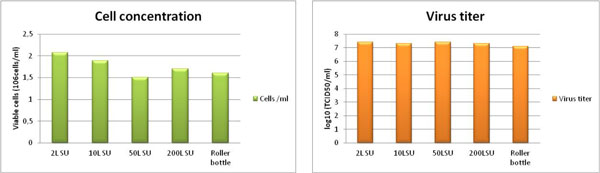
**Comparison of cell growth and virus production in 2 and 10L bioreactors and in a BIOSTAT®CultiBag STR 50 and 200L SU**.

## Conclusions

• Linear scalability from R&D to manufacturing.

• SU Bioreactors offer similar growth and productivity than conventional bioreactors or roller bottles but are more flexible.

• SU Bioreactors have a faster turnaround time between batches because there is much less cleaning and sterilizations.

• SU Bioreactors are the best solution when containment is required (BSL-3 laboratories).

• Comparable results between 2L, 10L, 50L and 200L:

 Cell density ⇒ Viability ⇒ Productivity ⇒ Product quality.

• Good agitation and easy speed calculation according scale-up.

• Bioreactor system allows even.

• Equivalent cell growth profiles.

• Equivalent cell population homogeneity on microcarriers. Good cell attachment and propagation.

Therefore offering a suitable process to produce our bovine vaccine using attachment dependent cells.

Next potential goal: Reduction COG

• Reducing number of RB needed to inoculate the bioreactor and increasing virus yields.

## Acknowledgements

Carme Aulinas, Elisabeth Cortés, Carme Torres and Xell Gratacós for the excellent laboratory work and Sartorius-Stedim biotech for technical support

